# Formulation and Characterization of Acetazolamide/Carvedilol Niosomal Gel for Glaucoma Treatment: In Vitro, and In Vivo Study

**DOI:** 10.3390/pharmaceutics13020221

**Published:** 2021-02-05

**Authors:** Rehab Abdelmonem, Sammar F. Elhabal, Nevine S. Abdelmalak, Mohamed A. El-Nabarawi, Mahmoud H. Teaima

**Affiliations:** 1 Department of Industrial Pharmacy, College of Pharmacy, Misr University for Science and Technology (MUST), 6th of October City, Giza 12566, Egypt; Rehab.abdelmonem@must.edu.eg; 2 Department of Pharmaceutics and Industrial Pharmacy, Faculty of Pharmacy, Modern University for Technology and Information (MTI), Mokattam, Cairo 11571, Egypt; 3 Department of Pharmaceutics and Industrial Pharmacy, Faculty of Pharmacy, Cairo University, Kasr El-Aini Street, Cairo 11562, Egypt; nevine.abdelmalak@pharma.cu.edu.eg (N.S.A.); mohamed.elnabarawi@pharma.cu.edu.eg (M.A.E.-N.); mahmoud.teaima@pharma.cu.edu.eg (M.H.T.); 4 Department of Pharmaceutics and Industrial Pharmacy, School of Pharmacy, Newgiza University (NGU), Km 22 Cairo-Alex Road, Giza 12256, Egypt

**Keywords:** acetazolamide, carvedilol, niosomes, gel, intraocular pressure, glaucoma

## Abstract

Acetazolamide (ACZ) is a diuretic used in glaucoma treatment; it has many side effects. Carvedilol (CAR) is a non-cardioselective beta-blocker used in the treatment of elevated intraocular pressure; it is subjected to the first-pass metabolism and causes fluids accumulation leading to edema. This study focuses on overcoming previous side effects by using a topical formula of a combination of the two previous drugs. Sixty formulations of niosomes containing Span 20, Span 60, Tween 20, and Tween 60 with two different ratios were prepared and characterized. Formulation with the lowest particle size (416.30 ± 0.23), the highest zeta potential (72.04 ± 0.43 mv), and the highest apparent coefficient of corneal permeability (0.02 ± 0.29 cm/h) were selected. The selected formula was incorporated into the gel using factorial design 2^3^. Niosomes (acetazolamide/carvedilol) consisting of Span 60 and cholesterol in the molar ratio (7:6), HMPC, and carbopol with two different ratios were used. The selected formula was subjected to an in vivo study of intraocular pressure in ocular hypertensive rabbits for 60 h. The sustained gel formula of the combination decreased (IOP) to normal after 1 h and sustained efficacy for 4 days. Histological analysis of rabbit eyeballs treated with the selected formula showed improvement in glaucomatous eye retinal atrophy.

## 1. Introduction

Glaucoma is a category of eye disease that may lead to vision loss caused by damage to the optic nerve and permanent blindness. It is characterized by a rise in intraocular pressure (IOP) resulting in changes in the fiber layer of the optic nerve and retinal nerve [1]. Open-angle glaucoma, in which the drainage angle of fluid inside the eye remains open, is the most prevalent type, with less common types like closed-angle glaucoma and normal-tension glaucoma [2]. Over time, open-angle glaucoma progresses slowly and no pain occurs. Peripheral vision, followed by central vision, can begin to decline, resulting in blindness if not treated. Closed-angle glaucoma can arise gradually or abruptly [3]. Extreme eye pressure, blurred vision, mid-dilated pupils, redness of the eye, and nausea can be involved in the sudden presentation. Vision loss from glaucoma is life long after it has occurred. Glaucoma-affected eyes are referred to as glaucomatous [4]. Acetazolamide (*N*-(5-Sulfamoyl-1,3,4-thiadiazol-2-yl) acetamide) (ACZ) is a diuretic inhibitor of carbon anhydrase (CA) with a half-life of removal after a single oral dose of approximately 2.4 to 5.7 h [5]. ACZ is used for the treatment of seizures, respiratory stimulants in patients with chronic obstructive pulmonary disease (COPD), glaucoma, intraocular pressure reduction, drug-induced edema, and heart failure-induced edema [6,7]. ACZ is included in the Biopharmaceutical Classification System (BCS) class IV, having poor solubility and low permeability of the membrane [8]. Carvedilol,(CAR)((±)-1-(carbazol-4-yloxy)-3-[[2-(methoxyphenoxy)methyl]amino]-2-propanol)anon-cardioselective beta-blocker antihypertensive drug. In addition, CAR is used for the prevention of hypertension, angina pectoris, heart failure, and for the treatment of elevated intraocular pressure [9,10]. CAR is rapidly removed from the gastrointestinal tract, but it is exposed to critical first-pass liver metabolism, so it is around 25 percent with poor absolute bioavailability. It has a short half-life of around 6–7 h [11] in plasma. CAR is included in the Biopharmaceutical Classification System (BCS) class II, with low solubility and poor bioavailability (around 25%) and high membrane permeability [12]. Because of the complex dissection of eye cell tissue designed to protect itself from harmful and toxic substances, most systems used to deliver the drug to the eye are not easy, making it difficult to link the drug with the necessary effective concentration [5]. The rapid and extensive precorneal loss caused by drainage, high tear fluid, and poor eye ability to resolve these problems is one of the key problems of conventional ophthalmic drug delivery systems, and an improvement in the contact time between the drug and the corneal surface is needed [13]. Because of prolonged eye contact, topical eye formulations are considered one of the best formulations, which increases the drug’s concentration in the cornea and releases the drug at an efficient concentration [14]. Several distinct benefits are provided by transdermal delivery systems. They avoid factors such as pH, enzymatic activity, and drug–food interactions that affect drug gastrointestinal absorption and pass the first-pass effect, providing continuous-release delivery for several days (useful for short-lived drugs with an elimination half-life) [15]. In this context, the production of eye drug delivery using a nanotechnology system such as niosomes, liposomes, micelles, dendrimers, microemulsion, nanoemulsion, various polymeric vesicles, and other nanoparticles was important [16]. Targeted drug delivery is directly to the eye where the therapeutic effect is needed and there is ease of administration, since it can be administered in liquid form, much like eye drops and topical eye gel solutions. The advantage of this technique drug used with low concentration due to having high permeability can penetrate ocular by transcellular or paracellular or a combination of them [17]. The current research focuses on investigating the topical efficacy of ACZ alone, CAR alone, and a mixture of ACZ and CAR for the treatment of glaucoma.

## 2. Materials and Methods

### 2.1. Drugs, Chemicals, and Reagent Kits

All used chemicals, solvents, and reagents were purchased and obtained from authorized sources and of analytical grade. Acetazolamide (ACZ) was purchased from Sigma-Aldrich Company, St. Louis, MO, USA. Carvedilol (CAR) was purchased from Hetero Drugs Company, Telangana, India, and supplied by Multipharma Pharmaceutical Company, Heliopolis, Cairo, Egypt. Polyoxyethylene (20) sorbitan monolaurate (Tween^®^ 20) was obtained from Nice Chemicals Private Limited Company, Kerala, India. Polyethylene glycol sorbitan monostearate (Tween^®^ 60) was obtained from Alpha Chemika Company, Mumbai, India. Sorbitan monolaurate (Span^®^ 20) and sorbitan monostearate (Span^®^ 60) were purchased from Oxford Laboratories Pvt. Ltd. Pharmaceutical Company, Mumbai, India. Cholesterol (95%), methanol, sodium acetate, and acetic acid were purchased from Sigma-Aldrich Company, St. Louis, MO, USA. Double distilled water and deionized water were generated through the spectra/pore dialysis membrane 12,000–14,000 molecular weight cut off (Spectrum Laboratories Inc., Rancho Dominguez, CA, USA) and cellulose nitrate membrane, a Millipore^®^ Filter 0.45 µm (Sartorius Stedim Biotech GmbH, Goettingen, Germany), hydroxypropylmethylcellulose (HPMC-E4) was obtained from (Tama, Tokyo, Japan); Carbopol was obtained from (Sigma Chemical Co., St. Louis, MO, USA); and have been used throughout the study.

### 2.2. Animals

Albino New Zealand rabbits with healthy eyes and without any diseases were obtained from the Animal House of Faculty of Pharmacy, Cairo University, Giza, Egypt. All animal housing and handling were conducted in compliance with the Research Ethics Committee (REC), Faculty of Pharmacy, Cairo University guidelines and following the research protocols recognized by the Animal Care Committee of the National Research Center (Cairo, Egypt) and were approved by the Ethics Committee (PI 2334 on 31 December 2018).

### 2.3. Formulation of Acetazolamide and Carvedilol Niosomes

Four types of non-ionic surfactants were used for the preparation of ACZ niosomes, CAR niosomes, and a combination of them namely Span 20, Span 60, Tween 20, and Tween 60. Cholesterol was added to all preparation. Two molar ratios of non-ionic surfactant and cholesterol were used, namely 7:6 and 7:4 [18,19]. The niosomal formulation was prepared using the reverse-phase evaporation technique [15]. The composition of niosomes formulations is illustrated in (Table 1) and the composition of combination niosomes formulations is illustrated in (Table 2).

### 2.4. Evaluation of the Prepared Acetazolamide and Carvedilol Niosomes

#### 2.4.1. Determination of Entrapment Efficiency (EE)

One milliliter of ACZ and CAR, respectively, was centrifuged with a cooling centrifuge at 20,000 rpm for 1 h at 4 °C (Sigma 3K 30, Osterode am Harz, Germany). The supernatant was isolated from the niosomal precipitate following the isolation of the niosomal formulation. The precipitated niosomal was washed (1 mL) with saline pH buffered with acetate (5.8) and re-centrifuged for 30 min to extract excess unentrapped. The combined supernatant was then diluted to (10 mL) with pH buffered by acetate (5.8). Using a UV-Vis spectrophotometer (Shimadzu UV 1650 Spectrophotometer, Kyoto, Japan) at λ max 265.8 and 286.4 nm, the concentration of unentrapped ACZ and CAR, respectively, was determined spectrophotometrically compared to the original drug added [20,21].

#### 2.4.2. Measurements of Particle Size, Distribution, and Zeta Potential (x)

The niosomal suspension of ACZ and CAR (100 μL) was diluted to 10 mL with bidistilled water, and then the particle size (PS) was measured using Zetasizer 3600(Malvern Instruments, Malvern, UK) by light scattering technique (DLS) technique at 25 °C. Zeta potential of ACZ and CAR niosomal suspension was measured by diluting an aliquot of the niosomes suspension with a large amount of bidistilled water, and evaluated for a surface charge at room temperature by using Zetasizer 3600. Three cycles were taken for each sample. ANOVA one-way statistical tests were applied to determine significant differences between drug particle size and zeta potential of formulae [22].

#### 2.4.3. Fourier Transform Infrared (FT-IR) Spectroscopy

Fourier transform infrared spectroscopy is commonly used to study small particles and molecules. This approach offers valuable knowledge about the three-dimensional structure data obtained from the diffraction of X-rays. The vibration frequencies of a given compound in FTIR are predicted in a specific region based on both the type of atoms and the type of chemical bonds present in the compound [23]. The FTIR spectrophotometer (Shimadzu 43000, Kyoto, Japan) was used to describe the chemically possible drug-polymer interactions. Briefly, using the KBr disk process, a pure CAR and ACZ compact disc and its electrospray samples were developed and evaluated at a scan range of 4000–400 cm^−1^ with a mean spectrum of 32 scans at a 2 cm^−1^ resolution [24].

#### 2.4.4. Differential Scanning Calorimetry (DSC) Analysis

On a Perkin-Elmer DSC, differential scanning calorimetry (DSC) analysis of the samples was carried out. Samples (6.5–10 mg) were heated on an aluminum pan under a nitrogen atmosphere at a heating rate of 100 °C/min over a temperature range of 50 to 3000 °C. The DSC study was performed under a 20 lb/in^2^ nitrogen gas flow [25].

#### 2.4.5. Morphology and Transmission Electron Microscopy (TEM)

A transmission electron microscope (TEM) (JEM-1230, Joel, Tokyo, Japan) was used to study the morphology of ACZ niosome, CAR niosome, and a mixture of niosome and niosome gel. Samples were mounted on the carbon-coated grid surface and negatively stained with 1% phosphotungstic acid aqueous solution and dried until visualization at room temperature [26].

#### 2.4.6. In Vitro Release Study of ACZ and CAR Loaded Nisosome

A modified Franz diffusion cell, as described above, was used to evaluate the in vitro release from the individual ACZ and CAR niosomes, their combination, and gel of this niosomes combination, and the solution of the previous formulation dispersion through a semi-permeable cellulose membrane Filter 0.45 µm was obtained from (Sartorius Stedim Biotech GmbH 37070, Goettingen, Germany). The in vitro profile of this formulation was studied to investigate the effect of the combination of niosome polymers and their gel on the ACZ and CAR in vitro release profiles.

### 2.5. Formulation of Acetazolamide and Carvedilol Niosomes Gel

Hydroxypropylmethylcellulose (HPMC-E4) and Carpobol respectively based combination ACZ and CAR niosomal gel were formulated in two ratios (2% and 4% *w*/*w*) to prepare HPMC-E4 gel, where the weighed amount of HPMC-E4 was dissolved gradually in acetate buffer pH (5.8) containing 0.5% combination niosomes by the aid of magnetic stirrer model 275 T, (Crest Ultrasonic Corp.; New York, NY, USA) at medium speed. Stirring was continued until the formation of gel base then left overnight for equilibration. Gel formulations were prepared by mixing the niosomal dispersions with the gels by a glass rod to have a final combination niosomes gel [27].

### 2.6. Evaluation of the Prepared Acetazolamide and Carvedilol Niosomes Gel

#### 2.6.1. Visual Inspection

The prepared formulae were examined for their physical characteristics such as color, clarity, and homogeneity.

#### 2.6.2. Determination of pH of the Prepared Acetazolamide and Carvedilol Niosomes Gels

The solution was prepared by dissolving 1 g of each gel formula in 9 g of distilled water using a magnetic stirrer (origin). The pH measurement using pH meter 3505–Jenway, Bibby Scientific Ltd., Dunmow, UK; was repeated three times for each formula and the average of the readings of three replicated was taken [28].

#### 2.6.3. Drug Content Studies

Dissolving the weighted sum of each gel mixture in around 20 mL methanol has determined the drug quality of gels. The resulting solutions were quantitatively transferred to volumetric flasks and sufficient dilutions were made of the same methanol solution. The resulting solutions were then purified by 0.45 μm membrane filters before the spectrophotometric analysis of the solution at 265.8 and 286.4 nm by the first spectrophotometric analysis of the derivative [29].

### 2.7. Evaluation of Rheological Properties of the Prepared Acetazolamide and Carvedilol Gels

The viscosity of the prepared niosomes gel was evaluated using a rotational cone and plate Brookfield viscometer (VIBRA Shinko, Deshi, Japan); at 25 ± 1 °C [30]. Approximately 2 g of the solution measured was applied on the plates, leaving the cone temperature of 25 ± 1 °C. The measurements were made over the 0.5 to 100 rpm range with 10 s between the two consecutive speeds, and then in a downward series.

The rheograms of the prepared formulae were plotted: the *y*-axis was taken to represent the shear rate (s^−1^) and the *x*-axis to represent the shear stress (dyne/cm^2^) and the viscosity centipoises (cp). The individual rheological data [ƞ min, ƞ max, Farrow’s constant (N)] for each of the tested gels were calculated and the hysteresis loop area was obtained. Rheological properties of thixotropic models can be investigated by hysteresis studies. The technique usually determines the areas between curve rheograms, corresponding to increasing shear rates and down curve rheogram obtained with decreasing shear rates following agitation times. The areas are in turn referred to as thixotropic areas. It is normally accepted that greater thixotropic area increased thixotropy [30]. The rheological data were analyzed by using Farrow’s equation [31] and power-law equation [32] to predict the rheological behavior of each formula.

Farrow’s equation:

Log D = N Log S − Log ƞ,(1)

where (d) shear velocity (dyne/cm^2^), (n) shear velocity (dyne/cm^2^), (n) (cp.) N (Farrow’s constant) is the slope of log D to log S, which reveals the deviation from the Newtonian flow. If N is below one, then dilatants flow is indicated (shear rate thickening). If N exceeds one, then the pseudoplastic flow is indicated (shear rate thinning).

Power law equation:

Ʈ = ƞ Ƴ^n^, (2)

where (Ʈ) shear stress (dyne/cm^2^), (ƞ) a constant called the consistency index (apparent viscosity), (Ƴ) shear rate (s^−1^), (n) power constant or flow index.

The slope of log shear stress Ʈ toward against log shear rate Ƴ equal n (power constant) which shows the deviation from the Newtonian flow. In the event of Newtonian behavior n = 1, whereas in the event of (shear thinning) 0 < n <1, while in the event of dilatants flow (shear-thickening) n > 1. All measurements were made in triplicate.

### 2.8. Ex Vivo Corneal Permeation Study

Ex vivo corneal permeation experiments with a diffusion area of 0.785 cm^2^ were performed using a modified Franz diffusion cell prepared. Fresh rabbit cornea was fixed between the donor and receptor compartments. Then, 100 μL of nisosmes dispersion, corresponding to 500 μg of ACZ and 500 μg of CAR and a combination of them, niosomes and niosomes gel were correctly measured in the donor cells. To ensure the sink state, the receptor compartment was filled with 10 mL of acetate buffer saline solution (pH 5.8) and held at 35 ± 1 °C under magnetic stirring at 100 rpm. Next, 0.5 mL of the permeation media was removed at the required time and an equivalent amount of fresh media was applied to the receptor cell. The samples were filtered and analyzed using a validated UV method through a 0.45 μm membrane. The ACZ and CAR solution mixture was used as a control and the results were recorded in three runs on average. In comparison with time, the quantity of drug permeating through the corneal epithelium was plotted, and the apparent coefficient of corneal permeability (cm/h).

The equation was determined:

Papp = Jss/C°,(3)

where Jss (steady stat flux) is the linear portion slope (μg/h·cm^2^) and C° is the initial drug concentration (μg/cm^2^) [33].

### 2.9. In Vivo Evaluation of the Combination of Acetazolamide and Carvedilol Niosomes

#### 2.9.1. Pharmacokinetic Study

Rabbits were randomly classified into five categories: the first category treated with ACZ niosome solution, the second category treated with CAR niosome, the third category treated with ACZ and CAR nisosome combination, the fourth category treated with ACZ and CAR solution combination, and the fifth category treated with ACZ nisosome combination. Throughout the procedure, the rabbits were held under anesthetic using sodium pentobarbital (30 mg/kg) injected into the marginal ear vein. After administering 100 μL of each formula individually, 100 μL of aqueous humor was extracted after 15, 30, 60, 120, 240, and 360 min with 1 mL of insulin needle and put in a centrifuge tube. Vortex combining with 0.5 mL of methanol precipitated the protein. The precipitated protein was extracted for 10 min at 10,000 rpm by centrifugation, and the drug concentration in the supernatant was determined by UV. Using WinNonlin pharmacokinetic software (Certara Inc., Princeton, NJ, USA), the pharmacokinetic parameters were determined using a noncompartmental process.

#### 2.9.2. Pharmacodynamic Study

The rabbits with paclitaxel eye drops were topically anesthetized. Dexamethasone (0.025 percent) was dissolved in saline and each eye was injected into the limb. Ophthalmotonometry (SW-500, Shanghai, China) was used to measure the IOP. The model was deemed good if the IOP was higher than the upper limit of the usual IOP of 24.4 mmHg and persisted for 1 week. Rabbits with high IOP were randomly assigned into five groups (six rabbits per group) where only the right eye of each rabbit was handled individually with each formula and considered as a fake control group and the left eye. For 4 days, the IOP was calculated and the results of lowering the IOP were compared.

#### 2.9.3. Histological Examination

After the pharmacokinetic and pharmacodynamic trials, the rabbits were sacrificed to a marginal vein by injecting phenobarbital sodium, then the eyeballs were removed and either 10% formalin was put. In ascending grades of alcohol, tissue specimens were cut, cleaned, and dehydrated in xylene, the dehydrated specimens were then cleared, enclosed in paraffin blocks, and sectioned at a thickness of 4–6 μm. The tissue parts obtained were deparaffinized with xylol and stained with hematoxylin and eosin (H&E) for histopathological analysis through the electric light microscope [34]. 

### 2.10. Statistical Analysis of Data

The one-way study of variance (ANOVA) test was used to investigate the substantial difference between the outcomes of tested formulas. The significance level was set at 0.05 and (*p* < 0.05).

## 3. Results and Discussion

All formulation prepared by using cholesterol was used with surfactants as an enhancer of niosomal membrane rigidity, as enhancer ability to cement the leaking space in the bilayer membranes to increase potency and efficacy of combination drug niosomes and their gel.

### 3.1. In Vitro Evaluation of Acetazolamide and Carvedilol Respectively

#### 3.1.1. Determination of Entrapment Efficiency (EE)

Entrapment efficiency (EE) is a fundamental characterization parameter that demonstrates the vesicle’s capacity for drug encapsulation. 

The quantity of initial drug (Wi) in suspension, the quantity of unloaded drug (Wf), and thus the quantity of loaded drug is determined using analytical methods including spectrophotometry UV according to the following equation:

EE percent = Wi − Wf/Wi = 100 percent,(4)

The parameters influencing the efficiency of entrapment are vesicle size, surfactant form, and cholesterol quantity. Alkyl chain length alters the permeability of the membrane, thereby impacting the performance of encapsulation. Compared to shorter alkyl chain surfactants, higher trapping efficiency is obtained with longer alkyl chain surfactants, niosomes obtained with Span 60 (C16) have a greater EE percentage than those obtained with Span 20 (C12) since Span 60 has the longest alkyl chain among them [35]. Similarly, Guinedi et al. prepared ACZ-loaded niosomes for the treatment of glaucoma and observed that in both the Rev and TFH methods, niosomes prepared using Span 60 had better EE% [18]. This was explained by the fact that Span 60 had an alkyl chain longer than Span 20. Increased cholesterol levels have been seen to cause entrapment efficiency at the first stage due to the binding of cholesterol to the hydrocarbon chains, which contributes to membrane stability. However, a further increase in the volume of cholesterol disrupts the bilayer structure and decreases the entrapment efficiency of niosomes [36,37]. Surfactant: cholesterol ratio decreased from 7:6 to 7:4 EE percent of ACZ, CAR load.

For niosomes that were prepared with Spans, as shown in (Table 3), all the entrapment efficiency results were above 90 percent. It is evident from the results that all formulations of niosomes prepared using Span 60 have an EE percent higher than those prepared using Span 20. This can be revealed that entrapment efficiency increases with an increase in the concentration and lipophilicity of surfactant and the results were ranked A+C-S60-C6) > A-S60-C6 and C-S60-C6 > A-S60-C4 and C-S60-C4 > A-S20-C6 and C-S20-C6 > A-S20-C4 and C-S20-C4 and the results were (97.91 ± 0.53%) > (97.75 ± 0.10% and 95.95 ± 0.13%) > (96.82 ± 0.18% and 95.64 ± 0.27%) > (93.53 ± 0.15% and 92.55 ± 0.25%) > (92.99 ± 0.77% and 92.43 ± 0.24%), respectively. Water-soluble surfactants, such as Span 20, Span 60 have improved eye bioavailability in niosomes because surfactants act as penetration enhancers that can strip the mucus layer and crack junctional complexes [38]. 

For niosomes prepared using Tweens, all the results of entrapment efficiency are above or equal to 90% as illustrated in (Table 3), indicating that the EE% of niosomes formulae with using Tween 20 was slightly higher than those found for niosomes formulae with using Tween 60, and the results were ranked A-T20-C4 and C-T20-C4, > A-T20-C6 and C-T20-C6, A-T60-C4 and C-T60-C4, > A-T60-C6 and C-T60-C6, and the results were (93.59 ± 0.53% and 93.14 ± 0.54%) > (91.03 ± 0.07% and 91.49 ± 0.32%) > (91.00 ± 0.23% and 91.57 ± 0.35%) > (90.54 ± 0.22% and 90.23 ± 0.06%), respectively.

#### 3.1.2. Measurements of Particle Size, Distribution, and Zeta Potential (x)

Vesicle (particle) size determination, the size of the vesicle is a significant parameter that influences the carrier’s biopharmaceutical role and ophthalmic formulation. The particle size of nanoparticles capable of penetrating through the cornea should typically be within range (400–777.56 nm) [39]. Patients handle smaller particles better than larger particles [40]. The most pivotal criteria for ocular delivery are particle size and uniform size distribution, as large-size particles can induce scratchy sensation, and PDIs are known to be the optimum quantity for healthy ocular delivery [41]. The increase in the concentration of polymers increased the viscosity of the organic phase. Consequently, it prevented the gradual dispersion of polymers in the aqueous phase. As a result, greater particle size was obtained for formulations with a high concentration of polymer. Transformers containing Span 60 have better encapsulation efficiency than those containing Span 20 and Tween 20, Tween 60, owing to higher lipophilicity, which in turn caused higher vesicle rigidity [42]. These results are consistent with other studies, such as those by Khalil et al. [43].

For niosomes prepared using Spans, the findings presented in (Table 3) showed that the particle diameter of the formulations of neutral charged ACZ and CAR niosomes with both molar ratios (7:6) and (7:4) using Span 20 (A-S20-C6 and C-S20-C6), > (A-S20-C4 and C-S20-C4), (628.95 ± 0.64 nm and 682.25 ± 0.34 nm) > (467.85 ± 0.92 nm and 481.75 ± 0.64 nm), was greater than the formulations of niosomes prepared with the corresponding aforementioned molar using Span 60 and the formulae were graded as being (A-S60-C4 and C-S60-C4), > (A-S60-C6 and C-S60-C6) and the results were (543.70 ± 0.98 nm and 477.25 ± 0.27 nm), > (419.85 ± 0.63 nm and 416.40 ± 0.48 nm), with statistically significant differences at level (*p* < 0.05).

For niosomes prepared using Tween, the formulas display the particle diameter of the formulations in (Table 3) as being (A-T60-C4 and C-T60-C4) > (A-T20-C4 and C-T20-C4), (A-T60-C6 and C-T60-C6) > (A-T20-C6 and C-T20-C6) and the results were (882.05 ± 0.34 nm and 840.65 ± 0.65 nm) > (877.40 ± 0.11 nm and 815.55 ± 0.75 nm), > (767.90 ± 0.85 nm and 732.60 ± 0.39 nm) > (749.55 nm ± 0.33 and 633.80 ± 0.84 nm), with statistically significant differences at level (*p* < 0.05).

Zeta potential is defined as the potential at the hydrodynamic. When analyzing the zeta potential to recognize the surface charges of the vesicles, it was observed that the zeta potential values of neutral charged niosomal formulations prepared using either the molar ratio of Span 20, Span 60, Tween 20, or Tween 60 decreased in the zone where the dispersion of the colloidal system is stable (range between (19.89 and −69.39 mV).

Zeta potential for niosomes prepared using Spans, showed the value of zeta potential of ACZ and CAR niosomes with both molar ratios (7:6) and (7:4) using Span 60 (A-S60-C6 and C-S60-C6), > (A-S60-C4 and C-S60-C4), (69.39 ± 0.77 mv and 61.1 ± 0.36 mv) > (50.94 ± 0.16 mv and 54.12 ± 0.80 mv), was greater than the formulations of niosomes prepared with the corresponding aforementioned molar using Span 20 and the formulae were graded as being (A-S20-C6 and C-S20-C6), > (A-S20-C4 and C-S20-C4) and the results were (43.17 ± 0.58 mv and 33.22 ± 0.09 mv), > (33.6 ± 0.58 mv and 39.33 ± 0.32 mv), with statistically significant differences at level (*p* < 0.05).

For niosomes prepared using Tween, showed the value of zeta potential of ACZ and CAR niosomes with both molar ratios (7:6) and (7:4) using Tween 20 (A-T20-C6 and C-T20-C6), > (A-T20-C4 and C-T20-C4), (28.54 ± 0.59 mv and 33.51 ± 0.65 mv) > (33.16 ± 0.88 mv and 23.49 ± 0.38 mv), was greater than the formulations of niosomes prepared with the corresponding aforementioned molar using Tween 60 and the formulae were graded as being (A-T60-C6 and C-T60-C6), > (A-T60-C4 and C-T60-C4) and the results were (31.5 ± 0.88 mv and 22 ± 0.04 mv), > (19.86 ± 0.54 mv and 37.92 ± 0.62 mv), with statistically significant differences at level (*p* < 0.05).

The values of zeta potential of niosomal vesicles are represented in (Table 3) the highest zeta potential was observed with Span 60 whereas the lowest zeta potential was in the case of Span 20, Tween 60, and Tween 20. This may be due to an increase in surfactant hydrophilicity; zeta potential also increases [44,45]. Large zeta potential values (positive or negative charges) reflect the stability of colloidal dispersions [46]. Zeta potential value of the optimized niosome formula A-S60-C6, C-S60-C6, and A+C-S60-C6 prepared by Span 60 has a 7:6 ratio of 69.39 ± 0.77 mv–61.1 ± 0.36 mv and 72.0 ± 0.43 mv, indicating the stability of the optimized transfersomal formulation. This stability can be due to the repulsion between the nanovesicles, which prevents their agglomeration, providing a suspension that is highly stable and evenly dispersed [47]. The above results indicated the formation of high physical stability uniform nanovesicles and the homogeneity of formulations and high physical stability uniform nanovesicles were established. while the best formulation according to the expert design of entrapment efficiency, particle size, and zeta potential parameter, formulation of acetazolamide and carvedilol prepared by using Span ratio 7:4 so used combination niosomes of them.

#### 3.1.3. Fourier Transforms Infrared (FT-IR) Spectroscopy Excipients

**Figure 1:**
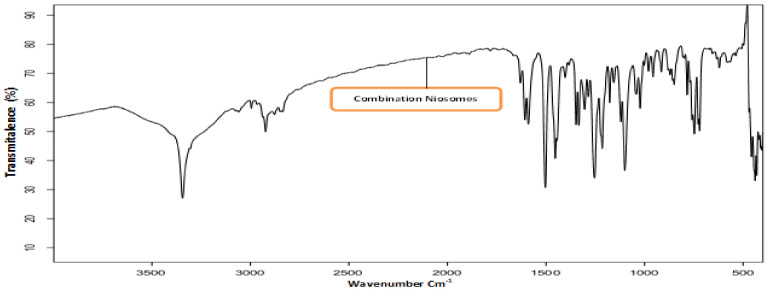
FTIR images of combination acetazolamide and carvedilol optimized transfersomal formula.

Drug-excipient interactions were analyzed using FTIR spectroscopy. The IR spectrum of ACZ confirmed the existence of its functional groups as follows: 3296.88 and 3174.70 cm^−1^ for the N-H stretching of the secondary amine. The presence of absorption at 1678.21 cm^−1^ was attributed to the carboxyl groups’ C=O stretching. The characteristic peak was at 1173.81 cm^−1^ due to the S=O stretch of sulfonyl groups. S-N stretching absorption was observed at 907.12 cm^−1^ [48]. FT-IR spectrum for CAR The FTIR CAR spectrum showed distinctive peaks at 3344.66 cm^−1^ (N-H and O-H stretch peaks merged), 2924.86 cm^−1^ (C-H stretch), 2840.56 cm^−1^ (C-O stretch), 1594.90 cm^−1^ (N-H bending vibrations), 1255.39 cm^−1^ (C-O stretching and O-H bending vibrations), and 1029.54 cm^−1^ (symmetric C-O-C stretching) [49,50,51]. The FTIR spectra of pure Eud also suggested peaks at 2991.35 cm^−1^ (CH aliphatic stretching) and 1732.64 cm^−1^ (-C=O stretching) [52,53]. Figure 1 shows the IR spectrum of optimized ACZ with Span 60 ratios 7:6 (F3), CAR Span 60 (F11) with the same ratio, and combination nisosmes of ACZ and CAR comprising Span 60 with a formula of 7:6 ratio (A+C-S20-C6) showing the characteristic peaks of ACZ and various excipients showing the absence of any chemical interactions between ACZ, CAR, and excipient.

#### 3.1.4. Differential Scanning Calorimetry (DSC) Analysis

**Figure 2:**
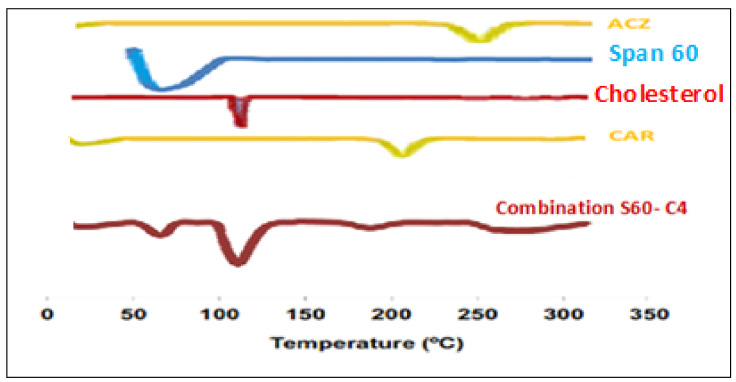
Differential scanning calorimetry (DSC) thermograms of pure ACZ, Span 60, cholesterol, CAR, and drug-loaded combination niosomal formulation.

The thermal behavior and the physical state of the drug could be detected through thermal analysis (DSC) is often used to examine the effect of drug loading on lipid particulate, to evaluate the state of the drug (crystalline, amorphous, molecularly dispersed) in the dispersions and to determine the extent of drug loading in the lipid carriers [54]. In addition, differential calorimetric scanning (DSC) is conducted to investigate the gel–liquid transition temperature of the niosomes [55]. Phase temperature is generally defined as the temperature that causes a change in the physical condition of the non-ionic surfactant or liquid from the phase, closely packed molecule, to liquid crystalline phase, loosely packed molecule, and fluid [56]. DSC analysis was carried out for selected formulation A+C-S60-C6 mixture of combination niosomes, CAR, ACZ, and Span 60 are presented in Figure 2 showed a sharp endothermic peak at 261 °C and 201 °C which corresponds to the melting of the drug. Span 60 has a peak observed between 50–60 °C indicated melting points of maximum excipients The cholesterol has the melting point of 148 °C which was shifted to 120 °C due to evaporation of water absorbed by the poloxamer during the formulation of combination nisosmes indicated the increase in phase transition temperature of niosomes upon loading with a combination of ACZ and CAR. The absence of the melting endotherm of combination suggested that the drug changed from crystalline to amorphous state. These results suggest significant interaction of a drug with the bilayer structure and can account for the enhanced entrapment of combination into niosomal formulations and sustained drug release this could be attributed to the perfect encapsulation of ACZ and CAR in the transfersomal vesicles and the dispersion of them as an amorphous state in the nanovesicles increasing the phase transition temperature [57]. The amorphous combination niosomes may be favorable due to the enhanced solubility of the active agent [58].

#### 3.1.5. Morphology and Transmission Electron Microscopy (TEM)

**Figure 3:**
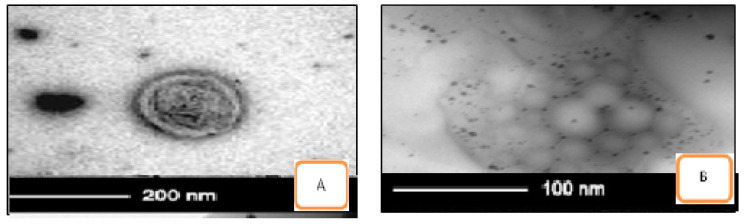
Transmission electron microscope (TEM) pictures of combination niosomes and niosomal gel (**A**) A+C-S60-C6 & (**B**) H-LV A+C-S60-C6.

**Figure 4:**
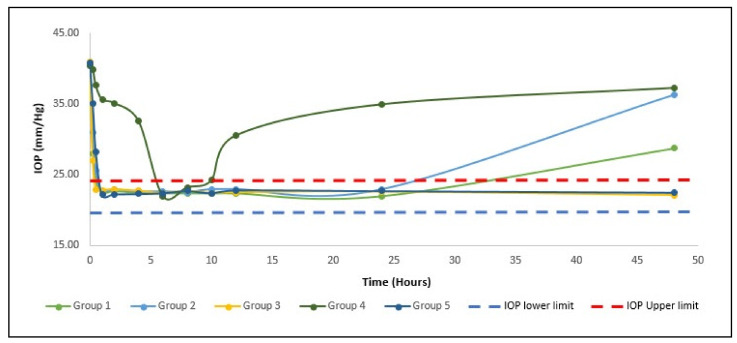
Mean IOP of hypertensive rabbits for first 48 h with a different administration in comparison with physiological minimum IOP for rabbit (IOP lower limit) and physiological maximum (IOP upper limit).

The photomicrographs TEM analysis confirmed that niosomal samples of ACZ and CAR are shown in Figure 3. It showed the presence of spherical and have a definite internal aqueous space homogenous population of unilamellar vesicles with closed bilayer vesicles structure consisting of a few concentric bilayers. The spherical morphology of the niosomal vesicles could be observed. Combination niosomes and their gel nisosmes of Span 60 and cholesterol with ratio 7:6, A+C-S60-C6 & H-LV A+C-S60-C6 showed higher percent drug entrapment and smaller particle size and high zeta potential. In vitro drug release and ocular permeation of different gel preparations showed sustained release and enhanced permeation compared to combination niosomes formulations containing the free drug. Among the niosomal gel formulations, HPMC gel with a low level showed the highest sustained release of the drug. The in vivo IOP of the selected combination niosomal and combination niosomal gel formulation was significantly lower pressure from 40.40 ± 1.10 to 22.15 ± 2.00 within 1 h and more sustained than the nisosmes individually and a corresponding non-niosomal formulation containing the free drug [21]. These results suggest that the combination noisome and combination niosomes containing HMPC-LV gels are promising formulations for sustained ocular delivery of ACZ and CAR Table 4 and Figure 4.

### 3.2. Evaluation of the Prepared ACZ and CAR Loaded Niosomes Gel Formulations

#### 3.2.1. Visual Inspection

Table 5 shows the physical properties and PH values of the freshly prepared formulae for the combination of ACZ and CAR loaded niosomes gels. All niosomal gels were evaluated for their clarity, turbidity, translucency, change in color, and precipitation. The physical examination of freshly prepared gels revealed that the formulae with high viscosity, except C-HV A+C-S60-C6 showed precipitation and H-HV A+C-S60-C6 showed highly viscous liquid. All gel formulae were homogeneous and white.

#### 3.2.2. Determination of pH of ACZ and CAR Loaded Niosomes Gel Formulations Study

As shown in Table 5, all combinations of ACZ and CAR niosomal gels exhibited values of pH between 5.64 and 5.92. These values are undoubtedly suitable and non-irritating to the eye [59,60].

#### 3.2.3. Drug Content of ACZ and CAR Loaded Niosomes Gel Formulations Study

Table 5 shows the average ACZ and CAR content in combination for selected formula prepared by Span 60 with two different ratios 7:6 and 7:4 of loaded niosomes HMPC and carbopol with low and high level with standard deviation, for the prepared formulae C-HV A+C-S60-C6 to H-LV A+C-S60-C6. The mean drug contents were in the acceptable range, in which respective drug content of ACZ and CAR were (99.10 ± 1.61%–98.80 ± 1.28%) and (100.75 ± 1.17–100.70 ± 2.05%) as mentioned in US pharmacopeia [61,62].

#### 3.2.4. Evaluation of Rheological Properties of the Prepared ACZ and CAR Loaded Niosomes Gel Formulations Study

The viscosity of a polymer solution is a measure of its resistance to flow, which is a complex function of its molecular weight, its concentration, as well as temperature and shear stress [63]. Rheological properties i.e., flow and viscoelastic properties of gels play an important role in the mixing and flow characteristics of materials, their packaging into containers [64], physical stability [65], and patients’ acceptability [66]. In addition, rheological properties influence drug release from semisolid formulations [67,68], its subsequent penetration into the eye, and also adhesive properties [65,67], i.e., the required contact time between the treated area and gel. The association between contact time and rheology for improved ophthalmic solution viscosity is readily understood. The shearing force on the preparation is high during blinking. If the viscosity at a high shear rate is too high (dilatant flow), this can irritate. If, on the other hand, the viscosity is too poor, there would be greater drainage. The viscosity at low shear should be high to limit the drainage between blinks (pseudoplastic flow). Non-Newtonian formulations that display pseudoplastic properties can acquire a viscosity decrease with an increased shear rate created by ocular movement. Pseudoplasticity is thus interesting because it offers significantly less resistance to blinking and shows much greater acceptance than viscous Newtonian formulations [69]. All combination ACZ and CAR loaded niosomes gel formulations revealed a non-Newtonian shear-thinning (pseudoplastic) flow behavior, where there is a decrease in viscosity by increase in the shear rate. The cause of shear-thinning flow may be due to progressive rupture of the internal structure of the formulations (by increasing shear) and its later reconstruction through Brownian movement [70]. Shear-thinning behavior is a desirable property for topical preparation since they should be thin during application and thick otherwise [71]. The rheological data obtained from imparting viscosity agents (polymers) based on all combination ACZ and CAR loaded niosomes gel formulations (C-HV A+C-S60-C6- H-LV A+C-S60-C6) were processed with Farrow’s equation and power-law equation. The Farrow’s constant (N) was greater than one and flow index (n) of the power-law equation was less than one upon increase polymer concentration, revealing a non–Newtonian shear-thinning pseudoplastic behavior as illustrated in Table 6. The pseudoplastic properties of combination ACZ and CAR loaded niosomes gel formulations. Besides, a non-thixotropic behavior was revealed, where the upcurve and down curve coincided or overlayed [72] except C-HV A+C-S60-C6 and C-LV A+C-S60-C6 prepared with carbopol HV and carbopol LV revealed a slightly thixotropic behavior. The non-thixotropic behavior indicated a time-independent flow; a property that helps the retention of gels on the eye. The thixotropic behavior indicated a time-dependent flow that requires enough time to reform again application [73]. From the power law, the flow index (n) values were <1 revealing a non-Newtonian shear thinning behavior. There was a significant decrease in flow index (n) upon increasing polymer concentration. Table 6 shows significantly increasing the viscosity at ƞ max (viscosity at the minimum shear rate) and ƞ min (viscosity at the maximum shear rate) by increase polymer concentration. The viscosity at ƞ max of ACZ and CAR loaded niosomes gel formulations (C-HV A+C-S60-C6- H-LV A+C-S60-C6) could be arranged as follows: C-LV A+C-S60-C6 > C-HV A+C-S60-C6 > H-HV A+C-S60-C6 > H-LV A+C-S60-C6.

### 3.3. Ex Vivo Corneal Permeation Study

The permeation parameters of acetazolamide and carvedilol niosomes and gel of them formulations through a membrane. The results revealed that A-S60-C6, C-S60-C6, and A+C-S60-C6, A+C-S60-C6 loaded to HMPC gel gave the best results through the flux (Jss) and the permeability coefficient (Kp). Most of the results revealed that the flux (Jss) and at 8 h was higher than the flux (Jss) at 24 h for niososmes and 96 hr for gel due to rapid permeation of the drug at the first phase during the eighth hours. Statistically, the results showed significant differences between the release extent of ACZ noisome, CAR niosomes, the combination of acetazolamide and carvedilol niososmes, and gel of them combination compared with combination solution in all formulae of niosomal at 8, 24, and 96 h at a significant level (*p* < 0.05) using one way ANOVA test, where (*p* = 3.73 × e^−13^), (*p* = 3.92 × e^−14^), (*p* = 6.888 × e^−9^), and (*p* = 2.55 × e^−6^), (*p* = 2.13 × e^−17^), respectively. This indicated that the incorporation of niosomal suspension into gel bases resulted in delayed permeation due to the presence of an additional diffusion barrier to the drug permeation [74]. The results of permeation parameters showed rapid drug permeation was observed during the initial phase up to 35% of ACZ and CAR permeated at the first eight hours in dissolution medium followed by a slowing in drug permeation, results illustrated in Table 6.

### 3.4. In-Vivo Evaluation of the Combination of Acetazolamide and Carvedilol Niosomes

#### 3.4.1. Pharmacokinetic Study

As shown in Table 7, drug concentration-time profiles and pharmacokinetic parameters were observed following a single installation. The first group (GP1) treated with ACZ niosomes showed the following results when comparing the results obtained from the five treated groups: the half-life (t½) was 3.31 ± 0.71 h and the area under the curve (AUC) from 0 to 8 h was 31.85 ± 2.70 μg h/mL with a peak concentration (Cmax) of 4.53 ± 0.52 μg/mL. The second group (GP2) treated with CAR niosomes had the following results: a half-life (t½) of 4.15 ± 0.51 h and a curve area (AUC) of 0 to 8 h of 31.99 ± 2.90 μg h/mL, a peak concentration (Cmax) of 3.98 ± 0.32 μg/mL. The third group (GP3) treated with ACZ and CAR combination niosomes had the following results: a half-life (t½) of 11.49 ± 0.93 h and the area under the curve (AUC) from 0 to 8 h was 79.59 ± 2.73 µg h/mL, peak concentration (Cmax) of 5.13 ± 0.52 µg/mL The results obtained from the fifth group (GP5) which was treated with combination ACZ and CAR niosomes gel with HPMC with low level showed the following parameters, AUC of 74.47 ± 1.72 µg h/mL, indicating a higher drug bioavailability and had a peak time (Tmax) of 60 min, which was shorter than that with combination ACZ and CAR solution indicating the rapid onset of action. Its Cmax was 6.52 ± 2.43 µg/mL, which was higher than that for CAR and ACZ solution (GP5), and had a t½ of 19.02 ± 0.87 h, which was 7.53 times longer than that of ACZ and CAR combination solution. These results were consistent with the higher transcorneal permeability combination and their gel compared to their solution. It can be concluded from the pharmacokinetic study that the combination gel was capable of prolonging the retention time of ACZ and CAR and enhance its bioavailability. These results are in agreement with several studies showed the effect of the encapsulation the drug into nanoparticles on drug pharmacokinetics in aqueous humor where Ban et al. found that dexamethasone charged lipid nanoparticle revealed higher drug retention time and enhanced drug permeation to the cornea and consequently higher ocular bioavailability in comparison with dexamethasone aqueous solution [75]. In addition, Hassan et al. found that leciplex nanoparticles are promising vectors to deliver the drug through ocular mucosa and releasing the drug in a sustained pattern in comparison with their solution [21].

#### 3.4.2. Pharmacodynamic Study

Five groups of New Zealand albino rabbits, each group comprised six rabbits. The usual eye pressure of 20.6–24.4 mmHg was assessed for 2 consecutive days [21]. The IOP was increased to above 40 mmHg after 2 weeks of injection of dexamethasone into the anterior chamber. During the measurement period, the IOP of the right eye of each rabbit in both groups remained above 40 mmHg, suggesting the absence of systemic drug absorption. For treated with ACZ and CAR niosomes (GP1 and GP2), respectively IOP decreased to normal (22.5 ± 2.0 mmHg) after 1 h of administration, IOP was retained within the normal range for twenty-four hours, then IOP increased steadily, as shown in (Figure 4). The IOP decreased to the usual value (22.6 ± 2.10 mmHg) for the combination niosomes ACZ and CAR treated group (GP3) after 30 min of administration and the IOP was retained within the normal range for 60 h, followed by the IOP. Where gel combination niosomes treated group (GP5) IOP reduced to normal after 1 h of administration, IOP was retained within the normal range for four days and then increase steadily. Gel formula has a shorter Tmax and longer retention time compared to its solution. The extent of % IOP reduction was statistically significant between group 3 and group 5 at the sixty hours and 4 days’ intervals (*p* < 0.05). This confirmed the sustained effect.

#### 3.4.3. Histological Examination

**Figure 5:**
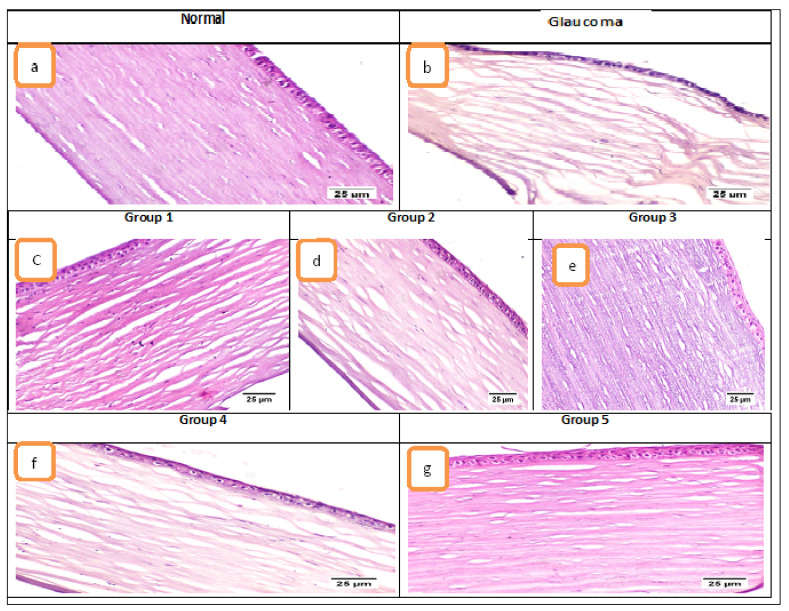
Photomicrographs of the cornea (H&E). Where (**a**) normal group showed histologically normal cornea, (**b**) glaucoma group showed marked corneal stromal edema, (**c**) Group 1 and (**d**) Group 2 showed mild edema, (**e**) Group 3 showed normal cornea, (**f**) Group 4 showed moderate edema, and (**g**) Group 5 showed normal cornea.

Microscopic examination of the eye’s tissue included the evaluation of the cornea, filtration angle, choroid, and retina. Concerning the cornea (Figure 5), the control normal sham group exhibited normal histological structure of cornea in which it consisted of five layers; (from outside to inside) non keratinized squamous epithelium, epithelial basement membrane, corneal stroma, Descemet’s membrane, and endothelium. The glaucoma group showed marked corneal edema manifested by dispersion of the corneal stroma by edematous fluid. Treatment using the different formulae resulted in improved corneal damage; Group 1 (ACZ niosomes contain Span 60 with ratio 7:6) and Group 2 (CAR niosomes contain Span 60 with ratio 7:6) showed mild corneal edema. Group 3 (combination niosomes of ACZ and CAR contain Span 60 with ratio 7:6) resulted in the significantly improved cornea, Group 4 (combination solution of ACZ and CAR) exhibited moderate corneal edema and Group 5 (the combination of ACZ and CAR niosomes gel) showed normal cornea. Regarding the filtration apparatus, in the normal group, the iridocorneal angle was containing a normally appearing trabecular meshwork that is responsible for the drainage of aqueous humor and the ciliary body which is a ring-shaped tissue located in the posterior ocular chamber it contains the ciliary muscle and a double layer of two, partly folded, neuro-epithelia, the non-pigmented, and the pigmented epithelium. The glaucomatous group showed extensive histopathological alterations in the filtration apparatus; the ciliary body showed marked thickening of the basement membrane with increased deposition of collagen and hyalinises of ciliary muscles. The trabecular meshwork showed a decrease in cellular components, increased matrix and fibrillary components, deposition of extracellular plaques, and hyalinization of the meshwork. The choroid is the blood vessel and connective tissue layer situated between the retina and sclera. The normal group showed normal choroid. The glaucoma group exhibited a compressed choroid. Group 1 and Group 2 treated groups exhibited mild choroidal compression and moderate with Group 4 while the choroid of Group 3 and Group 5 treated groups was histologically normal.

Retinal damage was obvious in the glaucoma group, diffuse retinal atrophy was observed with loss of inner ganglion cells. Few remaining ganglion cells were shrunken and hyperchromatic with pyknotic nuclei. Diffusely, there was variable thinning of the inner and outer plexiform nuclear layers. Microcystoid degeneration of the retina was noticed in which multiple clear round spaces were usually found in the inner plexiform layer. Group 1 treated group exhibited mild loss of ganglion cells and mild microcystin degeneration and moderate with Group 4 while the Group 2 treated group showed diffuse thinning of the retina. The retina of Group 3 and 5 treated groups was normal.

## 4. Conclusions

Our results indicate acetazolamide and carvedilol loaded niosomes were prepared using the reverse-phase evaporation technique for the treatment of glaucoma. Niosomes prepared by Span 60, Span 20, Tween 60, and Tween 60. The best formulation according to the expert design niososmes prepared by Span 60 and cholesterol with ratio 7:6 which decrease pressure of the eye rapidly and more significant. Synergism effect for treatment of glaucoma with a mixture of ACZ and CAR niosomes and combination gel of them noisome give the best results, the sustained effect of gel and significant cure of glaucoma disease due to a high ocular drug delivery device with no water retention side effect of β-blocker drugs, more bioavailability, and appropriate release kinetics. It can be concluded that ACZ and CAR formulated as combination niosomes and their gel form is a promising sustainable release formulation for the treatment of glaucoma, which can be used to enhance the patient’s compliance.

## Figures and Tables

**Table 1:** Composition of niosomes formulations, *n* = 3.

Formula Acetazolamide (ACZ)	Molar Ratio					Formula Carvedilol (CAR)
	Non-Ionic Surfactant				Cholesterol	
	Span 20	Span 60	Tween 20	Tween 60		
A-S20-C6	7	-	-	-	6	C-S20-C6
A-S20-C4	7	-	-	-	4	C-S20-C4
A-S60-C6	-	7	-	-	6	C-S60-C6
A-S60-C4	-	7	-	-	4	C-S60-C4
A-T20-C6	-	-	7	-	6	C-T20-C6
A-T20-C4	-	-	7	-	4	C-T20-C4
A-T60-C6	-	-	-	7	6	C-T60-C6
AT60-C4	-	-	-	7	4	C-T60-C4

**Table 2:** Composition of combination niosomes formulations, *n* = 3.

Formula Acetazolamide and Carvedilol	Molar Ratio				
	Non-Ionic Surfactant				Cholesterol
	Span 20	Span 60	Tween 20	Tween 60	
A+C-S20-C6	7	-	-	-	6
A+C-S20-C4	7	-	-	-	4
A+C-S60-C6	-	7	-	-	6
A+C-S60-C4	-	7	-	-	4
A+C-T20-C6	-	-	7	-	6
A+C-T20-C4	-	-	7	-	4
A+C-T60-C6	-	-	-	7	6
A+C-T60-C4	-	-	-	7	4

**Table 3:** Experimental runs, independent variables and measured responses, particle size, and zeta potential of acetazolamide and carvedilol niosomes formulae, *n* = 3.

Formula	A: Surfactant Type	B: Surfactant to CHO	Entrapment Efficiency (%EE)	Particle Size (nm)	Zeta Potential
A-S20-C6	Span 20	7:6	93.53 ± 0.15	628.95 ± 0.64	34.17 ± 0.58
C-S20-C6		7:6	92.55 ± 0.25	682.25 ± 0.34	33.22 ± 0.09
A-S20-C4		7:4	92.99 ± 0.77	467.85 ± 0.92	33.66 ± 0.58
C-S20-C4		7:4	92.43 ± 0.24	481.75 ± 0.64	39.33 ± 0.32
A-S60-C6	Span 60	7:6	97.75 ± 0.10	419.85 ± 0.63	69.39 ± 0.77
C-S60-C6		7:6	95.95 ± 0.13	416.40 ± 0.48	61.12 ± 0.36
A-S60-C4		7:4	96.82 ± 0.18	543.70 ± 0.98	50.94 ± 0.16
C-S60-C4		7:4	95.64 ± 0.27	477.25 ± 0.27	54.12 ± 0.80
A-T20-C6	Tween 20	7:6	91.03 ± 0.07	749.55 ± 0.33	28.54 ± 0.59
C-T20-C6		7:6	91.49 ± 0.32	633.80 ± 0.84	33.51 ± 0.65
A-T20-C4		7:4	93.59 ± 0.53	877.40 ± 0.11	33.16 ± 0.88
C-T20-C4		7:4	93.14 ± 0.54	815.55 ± 0.75	23.49 ± 0.38
A-T60-C6	Tween 60	7:6	90.54 ± 0.22	767.90 ± 0.85	31.15 ± 0.88
C-T60-C6		7:6	90.23 ± 0.06	732.60 ± 0.39	22.04 ± 0.33
A-T60-C4		7:4	91.00 ± 0.23	882.05 ± 0.34	19.86 ± 0.54
C-T60-C4		7:4	91.57 ± 0.35	840.65 ± 0.75	37.92 ± 0.61
A+C-S20-C6	Span 20	7:6	94.25 ± 0.33	459.59 ± 0.42	57.83 ± 0.34
A+C-S20-C4		7:4	94.53 ± 0.33	453.90 ± 0.35	58.73 ± 0.50
A+C-S60-C6	Span 60	7:6	97.91 ± 0.53	414.30 ± 0.23	72.04 ± 0.43
A+C-S60-C4		7:4	94.94 ± 0.06	434.21 ± 0.19	61.12 ± 0.70
A+C-T20-C6	Tween 20	7:6	92.51 ± 0.22	720.10 ± 0.53	43.31 ± 0.55
A+C-T20-C4		7:4	92.61 ± 0.37	757.30 ± 0.12	43.26 ± 0.32
A+C-T60-C6	Tween 60	7:6	91.90 ± 0.35	882.05 ± 0.34	39.26 ± 0.70
A+C-T60-C4		7:4	92.71 ± 0.45	840.65 ± 0.75	39.98 ± 0.55

Presented values are mean ± SD.

**Table 4:** Permeation parameters of in vitro release profiles of ACZ and CAR loaded niosomes formulations through membrane compare to their solution.

Niosomes	Mucoadhesive Polymer	Cumulative Amount Permeated/Area at 24 h (µg/cm^2^) mean ± SD	Flux (Jss) (µg/cm^2^/h)	Permeability Coefficient (Kp) (cm/h)	Lag Time (tlag) (min.)
A-S60-C6	-	607.96 ± 30.33	53.28 ± 0.04	0.01 ± 0.45	4.23 ± 0.31
C-S60-C6	-	523.04 ± 34.03	54.43 ± 8.21	0.02 ± 0.25	4.76 ± 0.82
A+C-S60-C6	-	706.17 ± 30.33	72.96 ± 1.01	0.01 ± 0.43	3.53 ± 0.39
H-LV A+C-S60-C6	HPMC-LV	1370.75 ± 85.12	145.02 ± 4.05	0.02 ± 0.29	2.53 ± 0.11
Combination ACZ and CAR Solution	-	393.07 ± 04.03	44.43 ± 4.21	0.02 ± 0.31	4.96 ± 0.92

Each value is an average of three determinations (*n* = 3).

**Table 5:** Physical properties and PH values of freshly prepared niosomes gel formulations study.

Mucoadhesive Polymer	Formula	Appearance Consistency	Colour	Homogeneity	Precipitation	PH (mean ± SD)	Average % ACZ & CAR Conc. (mean ± SD)
Carpobol-HV	C-HV A+C-S60-C6	Gel	White	Heterogeneous	Obvious	5.80 ± 0.77	99.10 ± 1.61 98.80 ± 1.28
Carpobol-LV	C-LV A+C-S60-C6	Gel	White	Homogenous	Nil	5.83 ± 0.06	97.04 ± 1.76 98.12 ± 1.52
HPMC-HV	H-HV A+C-S60-C6	Gel	White	Homogenous	Nil	5.83 ± 0.29	99.41 ± 1.46 99.02 ± 1.28
HPMC-LV	H-LV A+C-S60-C6	Gel	White	Homogenous	Nil	5.92 ± 0.12	100.75 ± 1.17 100.70 ± 2.05

**Table 6:** Rheological data and viscosity measurements of combination ACZ and CAR loaded niosomes gel formulations (*n* = 3).

Formula	Gelling Agent	% Conc.	Farrow’s Constant (N)	Flow Index (n)	Consistency Index (ƞ)	Flow Behavior	ƞ Max. at (Min. Shear Rate)	ƞ Min. at (Max. Shear Rate) (mean ± SD)
C-HV A+C-S60-C6	Carbopol-HV	4% ± 0.30	20.06 ± 0.13	0.05 ± 0.01	648.63 ± 0.51	Pseudoplastic	64,773.33 ± 130.51	402.73 ± 3.47
C-LV A+C-S60-C6	Carbopol-LV	2% ± 0.13	45.73 ± 0.15	0.02 ± 0.01	786.49 ± 0.57	Pseudoplastic	183,666.67 ± 288.68	907.83 ± 2.75
H-HV A+C-S60-C6	HPMC-HV	4% ± 0.14	1.37 ± 0.04	0.72 ± 0.07	120.23 ± 0.31	Pseudoplastic	9306 ± 3.61	523 ± 2.65
H-LV A+C-S60-C6	HPMC-LV	2% ± 0.13	1.63 ± 0.08	0.55 ± 0.02	28.31 ± 0.22	pseudoplastic	2734.67 ± 4.93	79.60 ± 1.00

**Table 7:** Pharmacokinetic study of in vitro release profiles of ACZ and CAR loaded niosomes formulations through membrane compare to their solution.

Parameters	GP1	GP2	GP3	GP4	GP5
t½ (h)	3.31 ± 0.71	4.15 ± 0.51	11.49 ± 0.93	2.73 ± 3.51	19.02 ± 0.87
tmax (min)	30.00 ± 1.80	30.00 ± 2.42	30.00 ± 2.22	60.00 ± 2.13	60.00 ± 2.10
Cmax (µg/mL)	4.53 ± 0.52	3.98 ± 0.32	5.13 ± 0.52	2.59 ± 2.23	6.52 ± 2.43
AUC (0–8) (µg h/mL)	31.85 ± 2.70	31.99 ± 2.90	79.59 ± 2.73	29.49 ± 1.92	74.47 ± 1.72

## Data Availability

Not applicable.
